# The Rare Medial-End Clavicle Fractures: Epidemiological Study on Inhabitants of a Suburban Area

**DOI:** 10.7759/cureus.18008

**Published:** 2021-09-15

**Authors:** Stefano Gumina, Stefano Carbone, Giuseppe Polizzotti, Carlo Paglialunga, Jacopo Preziosi Standoli, Vittorio Candela

**Affiliations:** 1 Orthopaedics and Traumatology, Sapienza University of Rome, Rome, ITA; 2 Orthopaedics, Sapienza University of Rome, Rome, ITA; 3 Orthopaedics and Traumatology, Sapienza University of Rome, Latina, ITA; 4 Medicine, Sapienza University of Rome, Rome, ITA; 5 Orthopedics and Traumatology, Sapienza University of Rome, Rome, ITA

**Keywords:** medial clavicle fracture, clavicle fracture, epidemiology of medial clavicle fractures, medial clavicle fractures demographics, mechanism of medial clavicle fracture

## Abstract

Background

Variable epidemiological data are known on medial clavicle fractures (MCFs).

Aim

To obtain demographic information regarding the etiopathogenesis of MCFs.

Materials and methods

All fractures were radiographically evaluated. Age; gender; side; date of fracture; fragment dislocation; associated fractures; fracture mechanism were collected. Three age groups were distinguished.

Results

1096 patients were enrolled: 29 (2.6%) had an MCF. Nineteen (66%) were males; mean age was 51.6 years (SD±24.4; range: 18-87). The right side was involved in 19 cases (66%). Nineteen fractures (66%) were un-displaced. Five patients (16.6%) had associated fractures.

Accidental falls represent the main cause of fracture. In advanced age (Group III), simple fall was the only cause of fracture. On the occasion of a fall, the right side was significantly more involved (p <0.05). Sports injuries were responsible for 22.2% of fractures, but for 42.9% of fractures in younger patients (Group I). Traffic accidents were responsible for five fractures (16.7%).

During the sunny seasons, the highest number of fractures occurred; the vast majority of fractures (83.3%) occurred on working days (p <0.05).

Conclusions

Medial clavicle fractures represent 2.6% of all clavicle fractures. Middle-aged males and the right side are more involved. Two-thirds of fractures are un-displaced. Accidental falls represent the main cause of fracture. During sunny seasons, the highest number of fractures occurred.

## Introduction

Fracture of the medial third of the clavicle is a rare event. It is equally known that these fractures mainly occur in middle-aged males following traffic accidents [1]. A recent systematic review, which collected data extrapolated from 17 studies, has shown that acute medial clavicle fractures are frequently associated with other traumas (mostly thoracic) that are extra-articular in 60% of cases and that the incidence of non-union is 5% [1].

However, very little data have emerged from epidemiological studies conducted on a considerable number of patients [2-8]. Furthermore, these data were taken from people who live in places with different degrees of urbanization, which have a great impact on the behavior, lifestyle, and customs of a community.

In addition, the normal anatomy of the clavicle is affected by morphological and morphometric differences, which might probably vary the least mechanical strength areas of the bone [9].

We have conducted an epidemiological study on a considerable sample of patients with clavicle fracture and inhabitants in a suburban area in order to obtain demographic information and data relating to the etiopathogenesis of medial clavicle fractures.

## Materials and methods

This studied cohort is constituted by all patients admitted to the Emergency Department of our hospital between January 1st, 2011 and April 1st, 2021, in a suburban area of Italy where live around 126,000 people. Patients with clavicular fractures were identified by using the International Statistical Classification of Diseases and Related Health Problems (ICD-10). All patients were clinically and radiographically evaluated within 24 hours from injury. Antero-posterior and 35° tilted radiographic (Zanca) views were used to classify the fractures. Patients aged under 18 years; with medial clavicle physeal injuries; with clavicle bifocal fracture and those whose fractures could not be well documented because the scarce quality of radiograms were excluded. Only permanent residents in the territory were included. The population consisted of mixed urban and rural people. Data relative to the last census indicate that the average age of the population is 43.8 years. All fractures were radiographically evaluated independently by two of the authors to confirm that the anatomical fracture pattern was correlated with the coding. If there was any disagreement between measurements, a consensus meeting with the senior author (SG) was undertaken to determine the most accurate value.

In agreement with Robinson’s classification, medial clavicle fractures have been considered those where the involved portion corresponded to the fifth of the bone lying medial to a vertical line drawn upwards from the center of the first rib [3,1]. Fractures were considered displaced when the displacement of the major fragments was greater than 100% [3]. Therefore, four groups of medial clavicle fracture were identified: Type 1A: extra-articular un-displaced fractures; Type 1B: extra-articular displaced fractures; Type 2A: intra-articular un-displaced fractures; Type 2B: intra-articular displaced fractures.

Clinical records of all patients were examined in order to collect information regarding age; gender; side; date of fracture; dislocation of fragments; associated fractures and eventual associated death. The causes of the fracture were grouped as: sports injury; simple fall; road-traffic accident; fall from height, and direct frontal blow. Three groups of patients were arbitrarily distinguished: those aged between 18 and 45 years (Group I); between 46 and 75 years (Group II), and >76 years (Group III).

We hereby certify that the manuscript entitled “The Rare Medial-End Clavicle Fractures: Epidemiological Study on Inhabitants of a Suburban Area” has been approved by the local IRB "ICOT Polo Pontino" with the approval number 12/2020.

## Results

A total of 1096 patients were enrolled for the study. Of these, 29 (2.6%) had a fracture of the medial end of the clavicle, 866 (79.0%) sustained a diaphyseal fracture, and 201 (18.4%) a lateral fracture. Nineteen of the 29 patients (66%) were male; 10 (34%) were female. The average age of the 29 patients was 51.6 years (SD±24.4; range 18-87). The right side was involved 19 times (66%).

Table 1 shows data relating to the demographic characteristics of the patients (age and gender), causes, and time at which the fracture occurred (season and day of the week) with respect to the three age groups (Groups I-III).

**Table 1:** Baseline characteristics of the studied group.

	Group I (18-45 y)		Group II (46-75 y)		Group III (>76 y)	
	M	F	M	F	M	F
A1	5 (50%)	0	1 (12.5%)	2 (50%)	1 (50%)	4 (100%)
A2	2 (20%)	1 (100%)	2 (25%)	1 (25%)	0	0
B1	2 (20%)	0	1 (12.5%)	1 (25%)	1 (50%)	0
B2	1 (10%)	0	4 (50%)	0	0	0
A1=Undisplaced extra articular fracture						
A2 = Undisplaced intra articular fracture						
B1= Displaced extra articular fracture						
B2= Displaced intra articular fracture						

Nineteen fractures (65.5%) were un-displaced. No statistically significant differences emerged by correlating gender and age with the type of fracture (un-displaced-displaced; intra-extra-articular).

Five (17.2%) of the 29 patients had associated fractures: four patients had multiple rib fractures and one pneumothorax; one had an ipsilateral humeral head fracture.

Accidental falls represent the main cause of fracture regardless of age. Patients who sustained a medial end clavicle fracture consequently to an accidental fall were significantly older (p<0.01).

**Figure 1:**
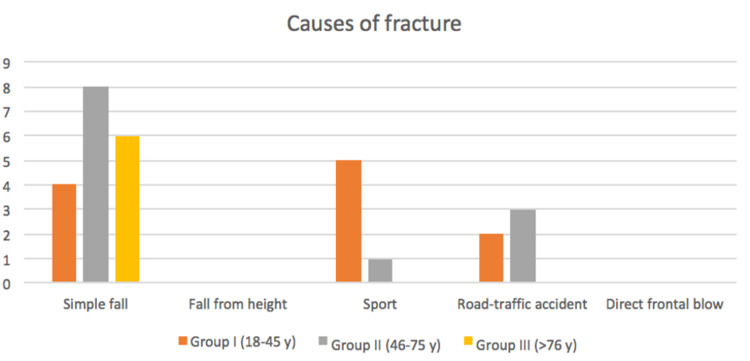
Mechanisms of trauma responsible for medial clavicle fracture; three groups according to age were distinguished. Fall from height and direct frontal blow did not cause medial clavicle fractures in our series.

In Group III, a simple fall was the only cause of the fracture (Figure 1). On the occasion of a fall, the possibility that the right side is more involved is statistically significant (p<0.05).

Considering the whole studied cohort, sports injuries are responsible for 20.6% of fractures, but for 45.5% of fractures in Group I. Significant differences were found between groups regarding frequency and cause of the fracture (p<0.05).

Road traffic accidents were responsible for five (17.2%) of the 29 fractures. None of our patients sustained a medial-end clavicle fracture following a fall from height or direct frontal blow.

**Figure 2:**
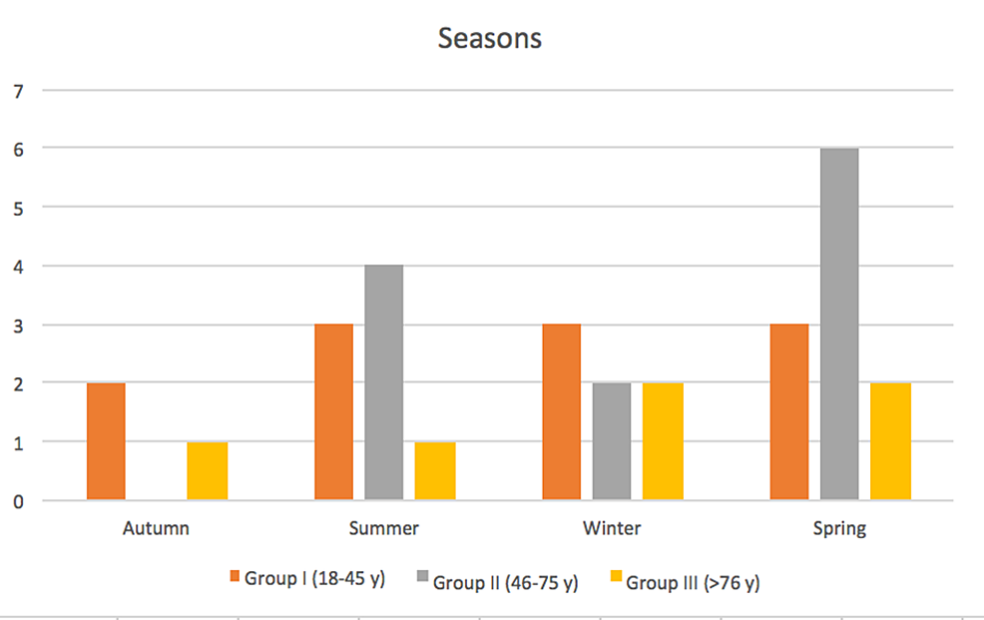
Distribution of the medial clavicle fractures according to seasons, in the three aged groups.

During the sunny seasons (spring-summer), the highest number of fractures occurred (19 fractures = 65.5%) (Figure 2).

**Figure 3:**
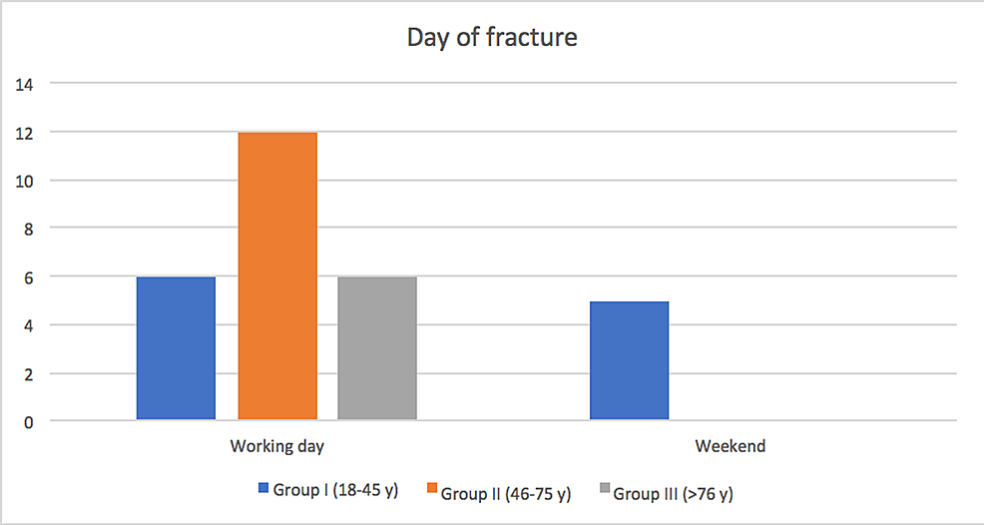
Distribution of medial clavicle fractures during the week in the three aged groups.

Only five (17.2%) of the 29 patients sustained fractures during the weekend. On working days, the average age of subjects who had medial-end clavicle fracture is significantly higher than that of patients who had fracture on the weekend (p<0.01) (Figure 3).

## Discussion

The medial clavicle fracture is historically considered rare. Therefore, data relating to the prevalence of this fracture can only be extrapolated from epidemiological studies conducted on a considerable sample of patients. Our study is among the few studies conducted on a cohort consisting of over 500 patients [1-5,7,8,10].

Literature indicates that the prevalence of the medial clavicle fracture ranges between 1.6% (Chinese population) and 15.4% (Australian population) [11,6]. The milestone studies on clavicle epidemiology report a prevalence of 4.5%, 3%, 2.8%, 2.6%, and 1.9%, respectively [7,2,3,4,8]. In the Throckmorton and Kuhn [5] series, the prevalence was 9.3%. Authors suspected that this high percentage might be attributed to the use of CT performed on all 593 patients. On the other hand, in the Salipas et al [6] series, consisting of 440 patients who underwent radiography only, the prevalence rises to 15.4%. It is plausible that these discrepancies are the consequence of the marked individual anatomical differences present in the clavicle. These differences refer to the different sinuosity of the lateral and medial curve [12]; clavicle index [9]; the presence of accessory joints [13], and the shape and spatial arrangement of the articular facets. De Palma [9], in a masterly monograph, described a definite relationship between the severity of the curves and the length of the bone. Therefore, the same traumatic event (for example a simple fall or a road traffic accident) can cause a clavicle fracture at different points of the bone depending on the sinuosity or straightness of the clavicle.

From a systematic review, relative to the medial clavicle fracture, emerged that males are involved in 78% of cases [1]. In our series, 66% of fractured patients were male. This is probably due to the greater involvement of young males in motor vehicle accidents. It is also plausible that males are more predisposed to have this type of fracture because they continue, over time, unlike females, to practice collision or contact sports or bike rides.

In the Throckmorton and Kuhn, Nordqvist et al, Robinson, and Salipas et al series, the mean age of patients with medial clavicle fracture was respectively 46, 51, 52, and 53 years [5,2,10,6]. In this series of patients, the average age was 51 years. These values are certainly higher than those that refer to the mean age of patients with clavicle fractures (without specifying the fracture site). In fact, Nordqvist et al, Postacchini et al, and Robinson encountered a mean age of 27, 29, and 33 years, respectively [2,4,3]. Therefore, it is plausible that the increase in the incidence of medial clavicle fracture in elderly persons may be related to the fact that the progressive senile osteopenia makes the trabecular bone of the clavicle end more prone to fracture.

A systematic review showed that the right side is involved in 46% of cases [1]. In this series of patients, two-thirds of patients reported a medial clavicle fracture of the right side. Since in this studied group the main cause of fracture was the simple fall, it is possible that the dominant side, usually the right one, was more involved because it was committed to minimizing the effects of the fall.

Although the Allman classification is largely used, it gives no guide to prognosis in terms of displacement, comminution or ligamentous injury [14-20], nor does it shows substantial to excellent levels of reliability and reproducibility among orthopedic trainees [3]. This could be one of the reasons why the percentage relating to medial clavicle fractures, classified as un-displaced according to Allman's classification had wide variability from 27% to 79% [4,2]. Another reason is strongly attributable to the studied population. In fact, our percentage of un-displaced fractures (66%) differs from that recorded by Herteleer et al (54%) and Robinson (82%), although we used Robinson’s classification system [8,3].

None of the patients included in this study had a medial clavicle fracture following a fall from height or direct frontal blow. Fifty-five percent of our patients sustained the fracture following a fall. This explains the small number of associated fractures (17.2%) and the absence, in the short and long term, of deaths related to the fracture. Our percentage relative to fractures due to a simple fall differs from what reported by Salipas et al (29%) [6]; Asadollahi and Bucknill (22%) [1]; Throckmorton and Kuhn (5.4%) [5]; in fact, they attribute to the motor vehicle accidents the responsibility of most of their fractures (54-84%). Again, these discrepancies are likely to depend on the demographic characteristics of the population to whom the studied groups belong. In fact, the community to which our patients belong is made up of people mainly dedicated to agriculture, pharmaceutical industry, and crafts and who are over 65 years of age in 21.4% of cases. Although it cannot be defined as a rural community, it is far from habits and lifestyles from that which inhabits large cities, and which suffers the effects of traffic and suburban degradation. This is demonstrated by the fact that in our series none were injured by gunshot wounds or were assaulted (8.7% in Throckmorton and Kuhn's series [5]) or sustained a fracture as the consequence of direct violence (14.3% in Robinson's series [3]).

In an epidemiological study on clavicular fractures, conducted during a two-year period in the late eighties and in a university town with a large proportion of student, Nowak et al observed that most fractures occurred during leisure time and outdoor activities [21]. This was reflected in the seasonal variation with fracture being more frequent during spring and summer but also in the distribution between different weekdays with an increase during the weekends. Unfortunately, from this excellent epidemiological work, it is not possible to extract data related to the seasonal and weekly distribution of the medial fractures. Two-thirds of the clavicle medial fractures occur during the sunny seasons (spring-summer) but only 17.2% of our studied group sustained the fracture during the weekend. Since our community exposed to fracture risk has an average age of 43.8 years and does not have an academic aptitude, the sunny seasons might only affect the number of injured patients following motorcycle or bicycle accidents. Furthermore, since our patients tend to be elderly and fractured following a fall, it is conceivable that the weekend exposes to a lower risk of fracture because in these days a family reunion can occur and this might facilitate the daily activities of the elderly patient, reducing the possibility that she/he may fall.

## Conclusions

Medial clavicle fractures are rare. In this series, consisting of 1096 patients with a clavicle fracture, the prevalence of medial fracture was 2.6%; the mean age was 51 years; 66% of the fractures were un-displaced. Males and the right side were more involved. Accidental falls represent the main cause of fracture. During the sunny seasons, the highest number of fractures occurred.

Data in the literature related to prevalence, middle age, percentage of un-displaced fractures, and causes of fracture are conflicting. This could be due to the poor reliability and reproducibility of the Allman classification, which is widely used, or could be specific to the studied population. In fact, the morphological and morphometric differences of the clavicle can make it more susceptible to fracture in different areas; demographic characteristics and different degrees of urbanization can affect life habits and, consequently, the possibility of fracture.
